# Modulation of vection latencies in the full-body illusion

**DOI:** 10.1371/journal.pone.0209189

**Published:** 2018-12-18

**Authors:** Alessandro Nesti, Giulio Rognini, Bruno Herbelin, Heinrich H. Bülthoff, Lewis Chuang, Olaf Blanke

**Affiliations:** 1 Department of Human Perception, Cognition and Action, Max Planck Institute for Biological Cybernetics, Tübingen, Germany; 2 Laboratory of Cognitive Neuroscience, Center for Neuroprosthetics and Brain Mind Institute, Swiss Federal Institute of Technology (EPFL), Geneva, Switzerland; Anglia Ruskin University, UNITED KINGDOM

## Abstract

Current neuroscientific models of bodily self-consciousness (BSC) argue that inaccurate integration of sensory signals leads to altered states of BSC. Indeed, using virtual reality technology, observers viewing a fake or virtual body while being exposed to tactile stimulation of the real body, can experience illusory ownership over–and mislocalization towards—the virtual body (Full-Body Illusion, FBI). Among the sensory inputs contributing to BSC, the vestibular system is believed to play a central role due to its importance in estimating self-motion and orientation. This theory is supported by clinical evidence that vestibular loss patients are more prone to altered BSC states, and by recent experimental evidence that visuo-vestibular conflicts can disrupt BSC in healthy individuals. Nevertheless, the contribution of vestibular information and self-motion perception to BSC remains largely unexplored. Here, we investigate the relationship between alterations of BSC and self-motion sensitivity in healthy individuals. Fifteen participants were exposed to visuo-vibrotactile conflicts designed to induce an FBI, and subsequently to visual rotations that evoked illusory self-motion (vection). We found that synchronous visuo-vibrotactile stimulation successfully induced the FBI, and further observed a relationship between the strength of the FBI and the time necessary for complete vection to arise. Specifically, higher self-reported FBI scores across synchronous and asynchronous conditions were associated to shorter vection latencies. Our findings are in agreement with clinical observations that vestibular loss patients have higher FBI susceptibility and lower vection latencies, and argue for increased visual over vestibular dependency during altered states of BSC.

## Introduction

In everyday life, we continuously and effortlessly integrate sensory information to create an internal representation of the environment and of our body. The latter is normally associated with a sense of occupying a given volume or location in space (self-location) within an owned body (self-identification), and contributes to the experience of a global “self” that is the subject of any given experience. Such sense of self, and its link to multisensory body representation, is commonly referred to as bodily self-consciousness (BSC) [1,2]. The crucial role of sensory cues (e.g., vision, touch, proprioception, interoception and vestibular signals) in shaping BSC is largely supported by clinical reports of altered BSC states. These typically result from electrical stimulation and/or neural damage localized in brain regions known to integrate multisensory bodily inputs [3–6]. Anatomical or functional lesions may, for instance, lead to ownership illusions of artificial limbs, or to autoscopic phenomena such as out-of-body experiences, where the world is experienced from a location outside of the physical body.

Recent experimental work demonstrated that altered states of BSC can be artificially induced in healthy individuals through systematic manipulations of sensory cues [7–9]. One commonly employed paradigm consists in providing participants with tactile stimulation of their back while they observe a fake or virtual body (avatar) being stroked in front of them (e.g., [7,8,10–15]). Vision of tactile stimulation *captures* the tactile sensation on the observers’ back (visual capture), and it has been argued that this leads to temporarily experiencing illusory ownership over—and mislocalization towards—the avatar. This bodily illusion mildly resembles the altered BSC symptoms typical of patients with autoscopic phenomena, and is generally referred to as full-body illusion (FBI).

The controlled induction of FBIs has been widely adopted as an experimental paradigm for the study of BSC in healthy individuals [7,8,10–15]. There are at least two reasons for this. First, it allows for the systematic bottom-up study of the stimulus characteristic and multisensory interactions that lead to abnormal states of BSC, including abnormal self-identification (which body ‘I’ identify with), self-location (where ‘I’ am located), and first-person perspective (from where ‘I’ experience the world) [1]. This is particularly relevant for developing comprehensive neurobiological models of self-consciousness [11] and relating abnormal alterations of BSC and their neural causes to altered states of BSC as induced in healthy subjects [1]. Second, FBI paradigms allow for investigating the consequences of systematic BSC alterations on perception and behaviour. For instance, it has been shown that the FBI affects multisensory processing [12,16,17], including increase in pain threshold [18,19], biases in perceived object sizes and distances [20,21], and shift of peripersonal space towards the avatar’s location [15]. In short, FBIs allow to study both how multisensory stimuli shape BSC, and how BSC impacts the way we perceive and process (multi)sensory stimuli. Therefore, this paradigm is generally accepted as a powerful tool for studying the multisensory processes underlying self-consciousness [2].

Compared to the large body of experimental work on the combination of visual with tactile, cardiac, auditory, proprioceptive and sensorimotor information [7,8,12,15–17,22,23], the vestibular contribution to BSC remains relatively unexplored. This is likely due to technological limitations and complexity in complementing the FBI with vestibular research. Nevertheless, the vestibular system is believed to significantly contribute to the neural computations underlying spatial aspects of BSC, due to its role in encoding self-motion and orientation, and due to the strong integrations of vestibular signals with motor, visual, somatosensory and proprioceptive signals [24–29]. This is further supported by the reports of illusory vestibular sensations, including illusory self-motion and illusory changes in self-location, in alterations of BSC caused by neurological diseases (i.e. out-of-body experiences and heauotscopic phenomena [30,31]). Similarly, patients with impaired or absent vestibular sensitivity are more inclined than healthy individuals to spontaneous [4] and experimentally induced [32] out-of-body experiences.

Additional evidence for the contribution of vestibular information in BSC comes from recent experiments showing that low-intensity electrical stimulation of the vestibular system can alter perceived ownership of body parts in neurological patients [33], and induce illusory body ownership in healthy participants [34]. Furthermore, similar electrical stimulations have been successfully employed to promote an embodied (i.e., first-person) perspective during a graphesthesia task [28]. More recently, Macauda and colleagues attempted to induce a visual-vestibular FBI by passively moving participants on a motion platform while they observed visual self-motion cues in virtual reality from a first person perspective [26]. Although FBI questionnaire scores were unaffected by manipulations of visuo-vestibular synchrony, they did report a modulation of skin temperature, a plausible index of changes in illusory body ownership [35] (but see also [36,37]). A different approach was adopted by Pfeiffer and colleagues [27], who manipulated visual gravity cues during a visuo-tactile FBI paradigm with participants lying supine on a bed. Interestingly, they report that asynchronous visuo-tactile stimulation was more frequently associated with an illusory downward-looking first-person perspective, suggesting that alterations of BSC can cause misperception of body orientation with respect to gravity. Altogether, these studies indicate an important contribution of vestibular cues in “anchoring” the self to the body, and that such link can be weakened using controlled manipulations of visuo-vestibular cues.

While these studies significantly contributed to better understanding the link between vestibular processing and BSC, it remains an open question whether alterations of BSC affect self-motion sensitivity, as it is the case for alterations of BSC on other perceptual phenomena such as size perception or pain perception (see above). Given the wide success of the FBI paradigm in altering BSC, and the subjective reports of drift in the FBI, one possibility would be to investigate changes in self-motion perception for healthy individuals experiencing the FBI. Self-motion sensitivity can be readily measured in a virtual reality setup by measuring the participants’ susceptibility to vection. Vection is an illusory perception of self-motion that can occur in a stationary observer exposed to large moving visual scenes, an intrinsically ambiguous stimulus that could result from either self-motion of the observer or object motion of the environment [38–41]. An often cited example is the feeling of motion when sitting on the train and watching the neighbouring train that begins to move. To solve this self- versus object motion ambiguity, the central nervous system relies on vestibular information, which only respond to linear and angular accelerations (including gravity) acting on the observer [42]. When the visual environment suddenly begins to move, the absence of a vestibular confirmatory signal causes the visual motion to be initially perceived as object motion. However, when the visual stimulus is sustained with constant velocity, the lack of inertial accelerations is no longer in conflict with the vestibular signal (which also report no inertial accelerations other than gravity). All the visual motion is then attributed to self-motion of the observer, who now perceives the visual environment as stationary [40]. Among the sensory and cognitive factors influencing the temporal dynamics of this transition (from object motion to self-motion perception), vestibular sensitivity has been shown to play a major role. Indeed, patients with vestibular loss have shorter vection onset times [43], and are overall more inclined towards experiencing vection [44]. Furthermore, vection latencies in healthy individuals negatively correlate with vestibular sensitivity [45], and are shorter in microgravity environments, indicating a shift towards higher reliance on visual, as opposed to vestibular, sensory cues [46,47]. Finally, an explicit dependency of vection latencies on vestibular sensitivity was proposed by Zacharias and Young [48] based on experimental data from heathy participants.

Here, we investigate the visual and vestibular contribution to BSC by studying the link between experimentally induced BSC alterations and the illusory perception of self-motion. Specifically, we address the question of whether the experience of the FBI is associated to changes in self-motion sensitivity. This is done by exploiting classical visuo-tactile conflicts to induce FBIs in heathy individuals, and then measuring their susceptibility to illusory self-motion perception (vection). The experimental design allows for multiple stimuli repetitions for each participant to better estimate their vection susceptibility and the effect of the visuo-tactile manipulation. We expect that the experimental setup can successfully induce the FBI (i.e., that participants report higher FBI scores when visuo-tactile stimuli are presented congruently). Furthermore, we expect a negative correlation between participants’ FBI ratings and their vection latencies, a result that would be consistent with the clinical reports of higher susceptibility to BSC alterations and illusory self-motion in vestibular loss patients.

## Methods

### Participants

Fifteen participants (age 21–35 years, six males) took part in the study. They were all naïve about the purpose of the study, they all had normal or corrected-to-normal vision and reported no history of balance disorders and motion sickness susceptibility. Participants gave their written informed consent (as outlined in PLOS consent form) prior to inclusion in the study, in accordance with the ethical standards specified by the 1964 Declaration of Helsinki, and received 30 CHF for compensation after having participated. The experiment was approved by the local ethical committee—La Commission d’Ethique de la Recherche Clinique de la Faculté et de Medicine de l’Université de Lausanne.

### Setup

**Fig 1:**
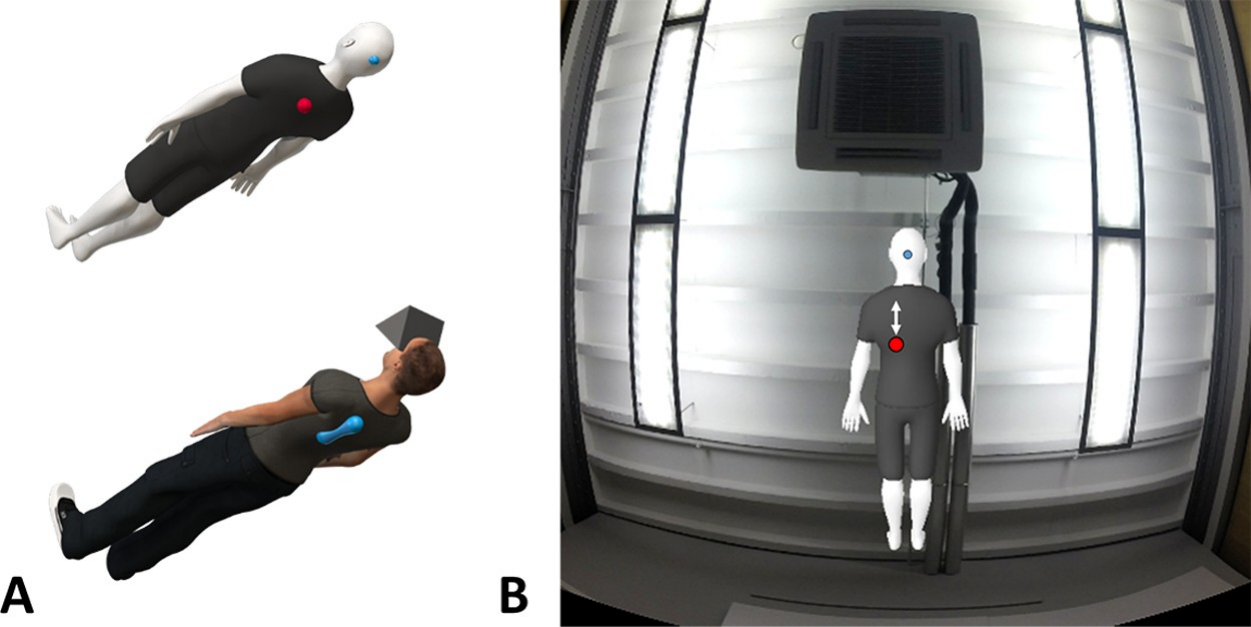
Experimental setup. A) Schematic representation of the experimental setup. Participants lying supine on a bed (bed not shown) received vibrotactile stimulation on their back (stimulator displayed in blue) and observed a virtual scene through an HMD. B) The visual environment consisted in a virtual replica of the experimental room, with a genderless avatar between the participants’ virtual position and the room ceiling. The scene further contained a blue fixation dot behind the avatar’s head and a red ball that could move on the avatar’s back along the trajectory indicated by the white arrow.

The experimental setup, depicted schematically in Fig 1A, was designed to induce alterations of BSC in healthy participants using visual and vibrotactile cues, and to occasionally induce vection.

Participants wore a black t-shirt provided by the experimenter, and a head mounted display (HMD) was used to present visual stimuli (Oculus Rift DK1, 640×800 pixels per eye, 4:5 aspect ratio, 110deg diagonal FOV, 60Hz refresh rate). For the entire duration of the experiment they laid supine on a bed, a position chosen to facilitate comparisons with previous studies on BSC and vestibular information processing [27,49], and to allow for possible follow-up studies in an fMRI scan. A custom-built vibrotactile stimulator [50] was taped by the experimenter on the participants back, parallel to the spine at approximately 10 cm to its left and 15 cm below the shoulder. The vibrotactile unit contained 6 aligned vibrators (PrecisionMicrodrives UK, model 310–118) and had an overall surface of 22.6 cm x 6 cm. Such fully wearable vibrotactile device allowed us to test supine participants without the need of a complex robotic system. Vibrators sounds could not be effectively masked, likely because of the bone property of conducting vibrations which can result in an acoustic percept [51]. We therefore installed a loudspeaker approximately 1.5 m above the participants’ trunk to play back the vibrators sound every time they were activated (see Stimuli and Discussion). Participants could control the progress of the experiment with a keyboard with three active buttons placed within reach of their right hand.

### Stimuli

Visual stimuli were created using Blender (Blender foundation) and consisted of a 3D rendered, genderless, grey-skinned avatar, seen from the back through the HMD (see Fig 1B). A virtual red ball was further included in the visual environment, and programmed to move vertically along the avatar’s back. In response to a trigger command, the red ball could perform either an upward or downward motion at constant velocity for 800 ms, travelling along a path that coincided with the position of the vibrotactile display on the participants’ back (see Fig 1). A blue fixation dot was also presented on the back of the avatar’s head. The background of the visual environment consisted of a picture of the ceiling of the experimental room that extended outside of the HMD field of view. Vection stimuli were occasionally presented during the experiment (see procedure) and consisted of a constant velocity rotation of the virtual ceiling at 15 deg/s around an axis intersecting both the participant’s and the avatar’s head (roll rotation), with a constant acceleration onset/offset lasting 2 s. For the entire duration of each vection stimulus, the avatar remained visible and did not rotate.

**Fig 2:**
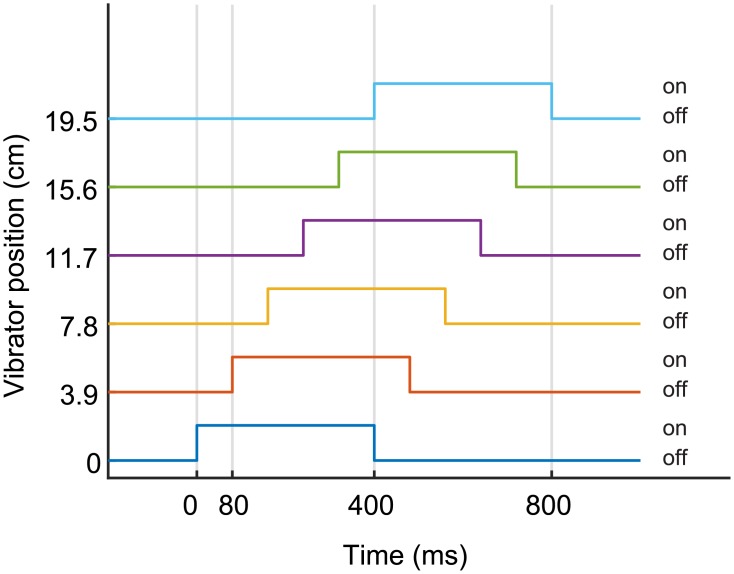
Activation profiles of the vibrators during the tactile illusion of apparent motion. Sequential activation profiles of the 6 vibrators of the vibrotactile unit during a vibrotactile illusion of a single apparent motion. A: Each vibrator is activated for 0.4 s, and a delay of 80 ms occurs between the activation of two adjacent vibrators. The resulting sensation is of a continuous movement lasting 0.8 s over a length of 19.5 cm along the participants’ back.

Vibrotactile stimuli were used to evoke in the participants the illusion of apparent motion [52–55], i.e., the illusion of a stimulation continuously moving between two or more discrete loci on the skin exposed to brief vibrotactile bursts. A vibrotactile stimulus consisted in the sequential activation of the 6 vibrators (Fig 2). Each vibrator remained active for 400 ms and a delay of 80 ms occurred between the activation of two adjacent vibrators, resulting in an overall apparent motion of 800 ms covering a length of 19.5 cm. Combining apparent movements and visual motion of the red ball allowed participants to experience a visuo-vibrotactile stimulus analogous to the visuo-tactile stroking commonly employed to induce FBI [7,8,15,16].

Sound cues from the vibrators could not be completely masked by active noise-cancelling headphones and white noise alone, likely due to the bone property of conducting sound (see also Discussion). Therefore, we used the speaker suspended above the participants to play back, in concomitance of every vibrotactile stimulus, a pre-recorded audio profile of the sound produced by the device during the vibrotactile stimulus (1000 Hz lowpass, fade-in and fade-out filtered).

In-house software (ExpyVR, http://lnco.epfl.ch/expyvr) was used for accurate real-time synchronization of visual, vibrotactile and auditory stimuli. The intrinsic delay between visual and vibrotactile stimulus onset was estimated to be approximately 100 ms, primarily caused by Bluetooth communication (~50 ms) and the vibrators mechanical time constant (~20 ms). The delay of the auditory stimulus was assessed prior to the experiment based on subjective reports from five participants, and found to be approximately 350 ms. To account for these delays, vibrotactile and auditory stimuli were triggered respectively 6 and 20 frames before the visual motion of the red ball, achieving synchronization accuracy < 17 ms.

A BSC questionnaire of four items was designed to elicit subjective responses to assess the induction of illusory bodily states (cf., [56]). The questionnaire consisted of four statements, listed in Table 1. The first three statements refer to key aspects of BSC, i.e., self-identification, touch referral and self-location respectively, while Q4 served as control for participants’ suggestibility. Statements were presented sequentially and in random order through the HMD against a black background, and responses were provided using two keys of the keyboard to adjust the position of a cursor along a continuous scale (100 steps, from “disagree” to “agree”).

**Table 1:** Questionnaire presented after each trial.

Q1	There were times when I felt as if the virtual body was my body
Q2	There were times when it seemed as if the red ball touching the virtual body was directly touching my body
Q3	There were times when I felt as if my body was drifting towards the virtual body
Q4	There were times when it seemed as if I might have three bodies.

### Procedure

Throughout the experiment, visuo-vibrotactile stimulation was intermingled with vection stimuli. Each trial started with a button press by the participants, who then performed a first baseline vection task in absence of vibrotactile stimulation. The entire vection task was performed while fixating the blue fixation point, and consisted in pressing any button of the keyboard as soon as participants confidently perceived the visual scene as stationary with respect to them, i.e. when they felt like all the motion they perceived was self-motion. The time between the onset of the vection stimulus and the button press (time to scene stationarity, TSS) was recorded, and the rotation of the virtual ceiling stopped. Upon self-terminating this baseline task, participants kept fixating the blue dot and experienced 60 s of visuo-vibrotactile stroking. Visuo-vibrotactile stimuli were interleaved by periods lasting between 1 and 3 s, during which no vibrotactile stimulation was delivered and the virtual red ball appeared stationary on the avatar’s back. Next, the visual background would again begin to rotate, signalling the participants to perform another vection task during which visuo-vibrotactile stimulation continued to be provided. After completing this second vection task, participants passively experienced 30 s of visuo-vibrotactile stroking, followed by a third vection task, followed by 30 s of visuo-vibrotactile stroking and a fourth and final vection task. At the end of each trial, participants completed the questionnaire. In total, each trial consisted therefore of four TSS measures and one questionnaire.

Participants were tested in a single experimental session composed of ten trials. In five trials, visual and vibrotactile stimuli were presented in synchrony (synchronous condition). In the remaining five trials, the visual motion of the virtual red ball was delayed by 500 ms with respect to the vibrotactile stimulus (asynchronous condition). Moreover, in the asynchronous condition the visual and vibrotactile stimuli moved in opposite direction, i.e., when the virtual red ball moved from the shoulder downwards, the apparent movement resembled upward motion along the vibrotactile display towards the shoulder, and vice versa. Prior to the experiment, a training session lasting approximately 5 minutes was performed to familiarize participants with the vection task, the apparent motion illusion and the questionnaire.

Overall, for each of the two conditions participants responded to the same questionnaire five times, and performed five baseline vection tasks and 15 (5x3) additional vection tasks, for a total of 10 questionnaires and 40 TSS measures per participant. Presentation order of synchronous and asynchronous trials, direction of the vection stimulus rotation (clockwise or counter-clockwise) and initial direction of the vibrotactile stroking (upward or downward) were fully randomized. Participants were required to make a break of 5 minutes after the 5^th^ trial and could take additional breaks before the start of each trial.

### Data analysis

Questionnaire scores were analysed to assess whether synchronous visuo-vibrotactile stroking led to higher questionnaire ratings than asynchronous stroking, in line with current theories on the multisensory origin of BSC and with a large pool of empirical studies [7–9,14,15]. For each statement of the questionnaire, we tested the effect of the synchrony factor (categorical, 2 levels: synchronous and asynchronous) in each participant (random factor) using a linear mixed-effects (LME) model:

$$q_{im}=\beta_{0}+\beta_{1}s_{im}+b_{0m}+b_{1m}s_{im}+\varepsilon_{im}$$ (1)

with i = 1,…N and m = 1,…M, where N the total number of trials, M represents the number of participants, q the questionnaire score, s the synchronicity level, epsilon is an error term, β_0_ and β_1_ are the fixed effect coefficients, and b_0m_ and b_1m_ are the random effect coefficients.

Although we did not expect a significant dependency of TSS on stimulus exposure time in the range of 1–5 minutes [57], we verified this using an LME model before averaging TSS over individual trials. Specifically, we tested the effect of stimulus onset time (continuous factor) and synchrony (categorical, 2 levels) on measured TSSs with the following model:

$${TSS}_{im}=\beta_{0}+\beta_{1}s_{im}+{\beta_{2}t_{im}+\beta_{3}s_{im}t_{im}+b}_{0m}+b_{1m}s_{im}+b_{2m}t_{im}+\varepsilon_{im}$$ (2)

where TSS are all the TSS excluding baselines, t is the time from the onset of the visual-vibrotactile stimulus to the onset of the corresponding vection task, and the other symbols are consistent in meaning with Eq 1.

Finally, we tested the hypothesis that a decrease in TSS values is associated to higher self-reported scores of BSC alterations. This was done by fitting, for each statement, an LME model of the form:

$${\bar{TSS}}_{im}=\beta_{0}+\beta_{1}s_{im}+{\beta_{2}q_{im}+\beta_{3}q_{im}s_{im}+b}_{0m}+b_{1m}s_{im}+\varepsilon_{im}$$ (3)

where overbar TSS are the average TSS values for each trial (baselines excluded) and the other symbols are consistent in meaning with Eq 1. Note that all models include a random factor to account for idiosyncrasies across participants.

Data analysis was performed in MATLAB (version R2016b, The MathWorks, Inc.) using the functions “fitlme()” and “anova()” from the Statistics and Machine Learning Toolbox, which allow to test whether the model coefficients associated to the different factors are different from 0. The need for transformation of the data prior to fitting the LME models was investigated based on visual inspection of the residuals. It was concluded that a square root transform was appropriate for the questionnaire scores and the TSS values to fulfil the assumption of homoscedasticity.

## Results

A total of 150 trials were completed, resulting in 150 questionnaires and 600 TSS measures. One trial (four TSS measures and one questionnaire) was discarded as the participant reported having lost concentration. Moreover, one TSS measure (0.47 s) was labelled as a mispress and another exceptionally long one as outlier (243 s, >10 standard deviations above the average TSS). Each of these two measures was replaced by the mean of the other two TSS measures of the same trial. The experiment lasted approximately 1 hour per participant, including a 5 minutes break after the 5^th^ trial. No participant reported symptoms of motion sickness or other forms of discomfort.

**Fig 3:**
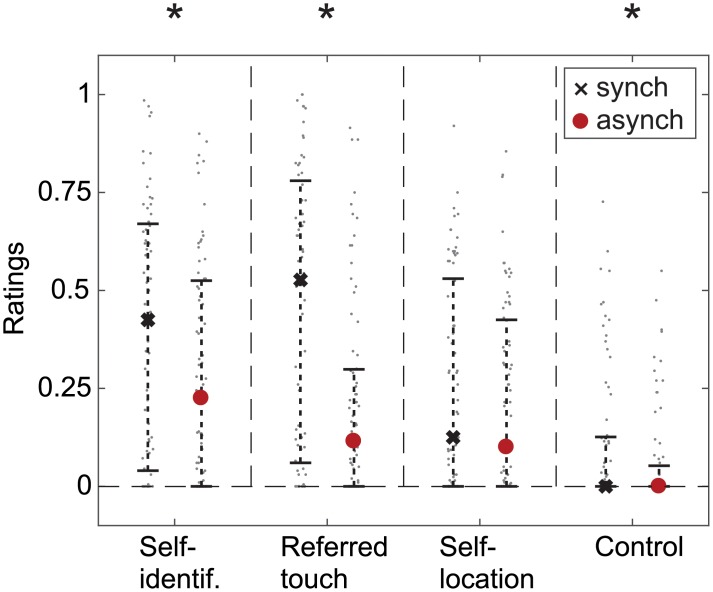
FBI questionnaire results. Median responses for the synchronous (black crosses) and asynchronous (red dots) conditions. Error bars show the interquartile range.

Collected ratings for the four statements of the FBI questionnaire are reported in Fig 3. Questionnaires analysis revealed a significant synchrony effect for Q1 (self-identification) and Q2 (touch referral), resulting in higher scores when participants experienced synchronous, as compared to asynchronous, visuo-vibrotactile stimuli (F(1,147) = 10.3, p = 0.002 and F(1,147) = 15.8, p < 0.001, respectively). No statistical difference was observed between Q3 (self-location) ratings during synchronous versus asynchronous stroking (F(1,147) = 3.3, p = 0.07). We further report a modulation of the control statement (Q4) by the synchrony of the visuo-vibrotactile stimuli (F(1,147) = 6.5, p = 0.01). Nevertheless, Q4 scores remained very low, with the median at 0 for synchronous and asynchronous conditions alike. Moreover, scores were overall significantly lower for Q4 than for Q1 (non-parametric Wilcoxon signed-rank test, Z = 8.08, p < 0.001), Q2 (Z = 7.38, p < 0.001) and Q3 (Z = 6.19, p<0.001), i.e., the three items that typically reflect the FBI. Overall, the questionnaires analysis revealed that the present setup can effectively induce the FBI.

In the analysis of the measured TSS, we found no significant interaction of stimulus synchrony and exposure time to the visuo-vibrotactile stimulus (F(1,443) = 1.60, p = 0.21). We further report no main effect of exposure time (F(1,444) = 1.05, p = 0.31) and no main effect of synchrony (F(1,444) = 0.16, p = 0.69). Therefore, TSS were averaged over trial for the subsequent analysis. TSSs during visuo-tactile stimulation were 24.20 s ± 18.18 s (mean ± standard deviation) and, as expected, were significantly lower (two-sample t-test: t(594) = 2.57, p = 0.01) than baseline TSS (28.88 ± 22.15 s).

**Fig 4:**
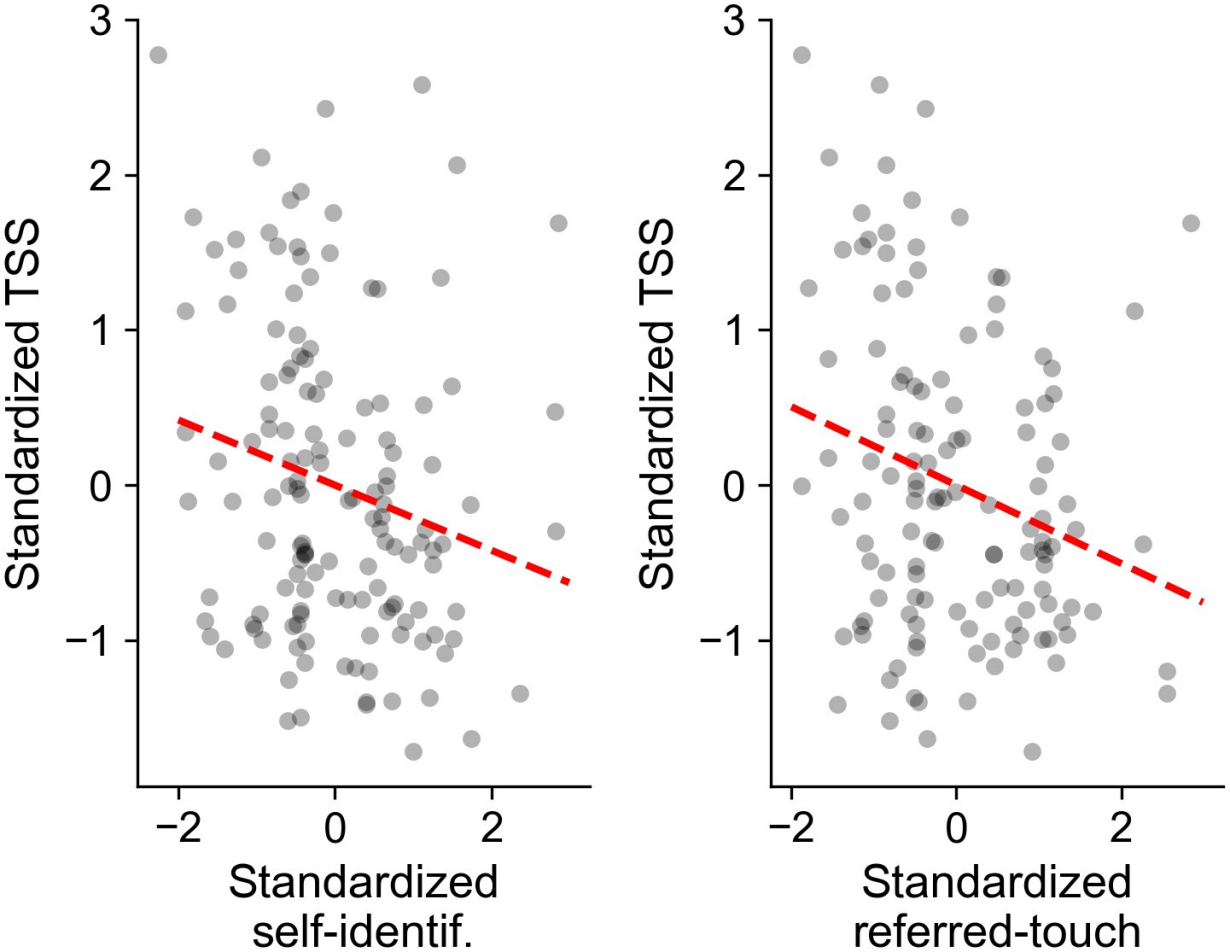
Relation between vection latencies and self-reported scores of self-identification and illusory touch referral. Measured TSS scores significantly decrease with increasing questionnaire scores on the extent of the bodily illusion. For graphical purposes, the plotted questionnaire scores and TSS measures are standardized to account for individual differences between participants.

Crucially, results show a significant relationship between TSSs and questionnaire ratings for Q1 and Q2 (F(1,146) = 5.3, p = 0.02 and F(1,146) = 13.3, p < 0.001, respectively), with no significant interaction between ratings and synchrony (F(1,145) < 0.57, p > 0.45) and no main effect of synchrony (F(1,146) < 3.3, p > 0.07) for any of the questionnaire statements. This indicates that shorter vection latencies are associated to higher self-reported self-identification with the avatar (Q1) and referred touch (Q2), independently of whether the FBI procedure was carried out in the synchronous or the asynchronous condition (Fig 4). No significant relationship was instead observed between Q3 (self-location) scores and TSSs (F(1,146) = 0.03, p = 0.86) nor between Q4 (control) and TSSs (F(1,146) = 0.14, p = 0.71). Finally, considering the significant effect of synchrony on Q4 scores, we further analysed the relationship between TSS and questionnaire scores (Q1 and Q2) to rule out possible suggestibility or compliance effects. This was done by subtracting, for each trial, the participant’s rating for Q4 from their ratings for Q1 and Q2. Applying the model from Eq 3 to the corrected scores resulted in identical conclusions (F(1,146) = 5.18, p = 0.02 and F(1,146) = 14.0, p <0.001 for Q1 and Q2 respectively).

For all models, we visually inspected the residuals and confirmed that all assumptions were fulfilled.

## Discussion

In the present study, we investigated vection latencies in heathy participants experiencing experimentally-induced alterations of BSC, as manifested in the form of the FBI, i.e., a bodily illusion resulting in misidentification and mislocalization of the bodily self. FBIs were induced using a novel experimental paradigm that combines virtual reality visual stimuli with a wearable haptic device that elicits the perceptual phenomenon of apparent motion. A total of 10 repetitions of the visuo-vibrotactile stimuli, and 40 vection stimuli, were presented to each participant to reliably asses the FBI and the participants’ vection latencies, quantified by their TSS. We found that the employed setup can successfully alter BSC, with higher FBI ratings reported during synchronous, as compared to asynchronous, visuo-vibrotactile stimulation. Moreover, self-reported ratings reflecting the strength of the experienced FBIs negatively correlated with vection latencies. In the next sections, we discuss in detail the novel experimental setup and the links between FBI, vection latencies and the interaction of visual and vestibular information.

### Visuo-vibrotactile FBI

Questionnaire data reported higher ratings in the synchronous as compared to the asynchronous condition for the self-identification and touch referral aspects of the FBI. Moreover, the difference in self-location scores between conditions approached statistical significance (a result that is not uncommon when using questionnaires rather than behavioural tasks to assess perceived self-location; see e.g., [16,35,58,59]). Scores in the control statement were very low, but nevertheless differed between conditions, a result which could indicate participants’ suggestibility. However, we find this possibility unlikely since ratings remained very low (medians are 0 in both conditions) and were significantly lower than any of the other FBI related statements. Overall, we believe that these results corroborate that the FBI can be more effectively evoked in participants experiencing tactile stimulation of their body while watching congruent tactile stimulation of an avatar, in line with previous literature on bodily illusions and neurocognitive models of BSC [2,10,11,60,61].

FBI scores in the present study appear slightly lower than in previously reported FBIs (cf. [8,15]). Although the large number of differences between experimental setups, designs and analysis procedures makes it hard to identify the causes, we speculate that an important role was played by both the use of a virtual avatar (rather than a video stream) and of a lying (rather than standing) participant’s position. Indeed, Salomon and colleagues [35] and Ionta and colleagues [49], who induced FBIs in supine participants using virtual reality, also report relatively lower scores. Moreover, in our study we replaced tactile stimuli with apparent motion illusions induced by vibrotactile stimulation, a change that could further contribute to reduce the FBI strength. Future studies should clarify the contribution of the individual sensory cues (e.g., visual, tactile, vestibular) and body posture to FBIs.

### Vection and visual-vestibular weighting during FBI

To the best of our knowledge, this is the first time that a vection task is combined with the experimental induction of the FBI to study the relationship between BSC and self-motion perception. Our first observation is that TSS measures after 60 s or more of visuo-vibrotactile stimulation were significantly lower than baseline TSSs measured before the onset of visual-vibrotactile stroking. This result is consistent with the inverse relationship between TSSs and bodily illusion ratings. Notably, this relationship was not affected by the synchrony of the visuo-vibrotactile stimuli. This result might seem counterintuitive at first, given the on average higher FBI ratings for synchronous stimuli. Note, however, that occasional instances of relatively high FBI scores during the asynchronous conditions (and similarly of relatively low FBI scores during the synchronous conditions) are not uncommon in our study (see Fig 3), as well as throughout the FBI literature. The wide range of FBI scores collected in both conditions allows our analysis to conclude that TSS measures decrease with increasing FBI ratings at a rate that is not affected by stimulus synchrony. This suggests that it is not the visuo-tactile timing of stimulation, but rather the strength of the induced subjective experience, that modulates vection latency.

A possible explanation for the observed inverse relationship between TSSs and FBI questionnaire scores could be a decrease in vestibular sensitivity during an FBI. Indeed, it is known that vestibular cues contribute to BSC [4,26,28,62], and that shorter vection latencies are associated with low vestibular sensitivity [43–47] (but see [63]). Taken together, these studies suggest that mild bodily illusion such as the FBI are associated with a weakening of the link between the self and the body, and in a decrease in vestibular sensitivity that facilitates vection. An alternative explanation is that, during the FBI, a higher weight is attributed to the visual over the vestibular channel. Visual dominance is commonly considered a major factor in bodily illusions [7–9], and higher weighting of visual cues is indeed expected to shorten vection latencies by helping solving the visual-vestibular conflict typical of vection stimuli [48,63] (see also Introduction). Naturally, the more general case of coexisting decreased vestibular sensitivity and increased visual weight is also possible. Finally, a possible effect of a vection stimulus on BSC, although seemingly unlikely, cannot be a priori discarded given the employed intermingled design. Future studies should further investigate the vestibular and visual contributions to the vection facilitation by developing experimental paradigms that separate the individual sensory components. One possibility could be the use of quantitative measures of unimodal perception such as motion detection thresholds [63].

### Methodological considerations

To complete the discussion of the main findings of this work, a series of methodological considerations are required, as important differences exists between earlier validated FBI setups and the stimuli employed in the present study.

First, stroking stimuli are generally delivered using a rod operated by either the experimenter [7–9,15,20,21] or a robot [14,27]. While vibrotactile stimuli have been previously employed to induce the FBI [13], our study is, to the best of our knowledge, the first to employ apparent motion illusions in an FBI setup. All participants reported, prior to the experiment, a convincing motion sensation during vibrotactile stimuli. Moreover, the effectiveness of the setup in inducing FBIs is supported by the questionnaires and TSSs analysis (see also subsection “Visual-vibrotactile FBI”).

A further novelty consisted in the use of auditory cues: because pilot sessions revealed that complete masking of the vibrators sound was not possible, we found it necessary to address the possibility that the inferential processes underlying BSC could include available auditory cues (localized at the participants’ back). In an attempt to overcome such possible biases, playback of the vibrators sound was provided through a speaker suspended above the participants (i.e., where the visual avatar was presented). While this measure might not completely rule out an auditory influence, we find it unlikely that it could affect the main conclusions of the study since auditory and vibrotactile stimuli were always presented synchronously. The role of auditory cues and trimodal sensory interactions in shaping BSC is a relatively unexplored research direction that certainly deserves further attentions [17,64].

In the present study, we further combined a classical FBI experimental design with a vection task. To evoke vection, we used constant velocity visual rotations, ramp onsets/offsets and a naturalistic virtual environment. These widely employed stimulus properties minimize visual-vestibular sensory conflicts and favours a coherent self-motion perception [38,65,66] (see also Introduction). Testing roll vection in participants lying on their back was necessary to prevent conflicts with the perceived gravity vector and, at the same time, provide rotations around an axis intersecting both the participant’s and the avatar’s head. This ensured that the avatar remained in the centre of the participants’ visual field. Average TSSs were approximately 25 s, in line with previous literature reporting vection onset times ranging between 2 and 40 s [41,65], with high inter-individual variability.

Finally, due to the fluctuating nature of bodily illusions, we decided to maintain visuo-vibrotactile stimulation throughout the vection task. Note that this inevitably results in longer periods of visuo-vibrotactile stimulations during trials where vection took longer to arise, hence raising the question of whether sustained vibrotactile stimulation (i.e., longer than at least 60 s) could systematically affect FBI scores. This possibility seems unlikely, based on previous reports of constant proprioceptive drifts during a related alteration of BSC, the rubber hand illusion, for stimulation periods longer than 40 s [57]. Consistently, we observed no significant relationship between TTS measures and the exposure time to visuo-vibrotactile stroking. Furthermore, we note that if FBI scores were to depend on exposure time, one would expect a positive relationship between FBI scores and the duration of vection stimuli (i.e., the TSS measures), whereas our data show the opposite trend.

### Conclusion

In this work, we studied vection susceptibility under conditions of altered BSC, experimentally induced in healthy participants using a modified vibrotactile FBI paradigm. We found that when participants reported high FBI scores (body ownership, illusory touch) they also showed increased susceptibility to vection, as quantified by shorter vection latencies (TSSs). These results suggest that mild BSC alterations are associated with changes in the neural integration processes of visuo-vestibular cues for self-motion perception.

The employed novel setup and methodology proved to be well-suited to study bodily illusions, and sets a useful precedent for employing a vection task to complement subjective self-reports with behavioural measurements. Additionally, the use of wearable technologies and the full automatization of stimuli and data collection routines allows for precise and reproducible visuo-vibrotactile manipulations and repeated stimulations, and opens new possibilities for studying BSC during more ecological scenarios, including locomotion.

## Supporting information

S1 Data Raw data. This folder contains the raw data collected during the study.
(ZIP)
